# Mortality and Predictors of Mortality Among COVID-19 Patients in Kiambu County, Kenya

**DOI:** 10.3390/covid5060076

**Published:** 2025-05-23

**Authors:** Teresia Njoki Kimani, Nyamai Mutono, Anita Makori, Patricia Mumbua Wambua, Patrick Nyaga, Jesse Gitaka, Omu Anzala, Samuel M. Thumbi

**Affiliations:** 1 Department of Medical Microbiology & Immunology, University of Nairobi, Nairobi 00200, Kenya; 2 Paul G Allen School for Global Health, Washington State University, Pullman, WA 99164, USA; 3 Centre for Epidemiological Modelling and Analysis, University of Nairobi, Nairobi 00200, Kenya; 4 Department of Health, County Government of Kiambu, Kiambu County 00900, Kenya; 5 Tigoni Level 4 Hospital, Kiambu County 00900, Kenya; 6 Centre for Research in Infectious Disease, Mount Kenya University, Thika 01000, Kenya; 7 KAVI-Institute of Clinical Research, University of Nairobi, Nairobi 00200, Kenya; 8 Institute of Immunology and Infection Research, University of Edinburgh, Edinburgh EH9 3JT, UK

**Keywords:** SARS-CoV-2, COVID-19, mortality, survival analysis, Africa

## Abstract

SARS-CoV-2 continues to circulate with new variants of uncertain transmissibility and virulence arising over time and resulting in varying morbidity and mortality between and within countries. This study aimed to identify the predictors of mortality among hospitalized COVID-19 patients across the first five waves of the pandemic. We conducted a retrospective cohort study at Tigoni Level 4 Hospital in Kenya. The study included patients admitted between June 2020 to August 2022 who tested positive for SARS-CoV-2. Sociodemographic and clinical data were abstracted from patient records at the time of admission and throughout their hospital stay. We employed Cox proportional hazard regression analysis to estimate the time to event (discharge or death) and identify predictors of mortality. Both time-varying and non-time-varying covariates were included in the models. A total of 1985 patients were admitted, of whom 557 (28%) died. The median hospital stay was 4 (1.0–8.0) days and 9 (5.0–13.0) days for patients who died and those who were discharged alive, respectively. Compared to patients admitted during wave 1, those admitted during the subsequent waves had high risk of death estimated at adjusted HR: 1.66 (95% CI 1.2, 2.54), 5.17 (95% CI 3.55, 7.53), 2.62 (95% CI 1.87, 3.67), and 2.17 (95% CI 1.51, 3.11) for waves 2, 3, 4, and 5, respectively. A proportion of patients presented with persistent chest pain, cough, and hypoxia and continued oxygen therapy for more than two months. In addition, patients who had persistent fever, hypoxia, cough, and fatigue had a significant mortality risk (adjusted HR: 3.00; 95% CI: 1.81–4.98; HR: 1.97; 95% CI: 1.73–2.26; HR: 1.47; 95% CI: 1.24–1.75; HR: 1.64; 95% CI: 1.05–2.54). Conversely, patients who had low oxygen saturation and received oxygen at admission had a 76% (HR: 0.24; 95% CI: 0.13–0.42) reduction in mortality risk and in addition patients whose treatment was altered had a 49% reduction in mortality risk (HR: 0.51; CI: 0.45–0.58). Our study highlights the benefits of oxygen therapy on the outcome of COVID-19 patients and justifies the need to increase investments in oxygen especially in low-and-middle-income countries. It also confirms the need to analyze the pandemic by the different waves.

## 1. Introduction

In the aftermath of the global pandemic, it is crucial to learn from the challenges posed by SARS-CoV-2. Over four years since the first COVID-19 case, the pandemic’s evolution in Africa remains less understood. As of May 2024, Africa reported 13.14 million cases and 18.16 cumulative excess deaths per 100,000 people. This represents only 5.2% and 12.7% of the cases reported in Europe and the United States, respectively [1]. In stark contrast, Black Americans with African ancestry have disproportionately accounted for a significant share of COVID-19 cases and deaths in the United States [2]. Despite multiple SARS-CoV-2 seroprevalence studies in Africa revealing high community exposure, reported cases and deaths do not mirror these findings [3–6]. This under ascertainment of COVID-19 cases in Africa, estimated at a factor of 8.5, is attributed to limited testing capacities and weak surveillance and health systems [7]. Additionally, the low morbidity and mortality in the continent was hypothesized to be due to prior exposure to cross-reactive viruses, youthful population, and favorable weather [8].

Globally, the virus rapidly adapted, with resultant fluctuating peaks in cases, hospitalizations, and deaths. Since Kenya’s first reported case on 13 March 2020, the pandemic has occurred in five distinct waves, each associated with unique predominant genetic lineages [9]. The initial peaks in August (wave 1) and November 2020 (wave 2) were dominated by the B.1 lineage. The third peak in April 2021 (wave 3) was driven by the Alpha variant. The Delta variant contributed to the largest peak in cases and deaths observed in August 2021 (wave 4), while the Omicron variant drove the fifth wave that peaked in mid-January 2022 (wave 5) [10,11]. These changing epidemiological patterns posed significant challenges to the health system, particularly in allocating and repurposing limited resources leading to significant disruption in provision and access to essential health services [12]. This strain in the health system necessitated innovative approaches to ensure continuity of essential health services, such as dedicating selected facilities for management of COVID-19 patients.

The clinical spectrum of SARS-CoV-2 infection varied, and patients experienced a range of clinical manifestations from asymptomatic, mild to moderate, severe, and critical illness [13]. Patients who presented with asymptomatic or mild disease were managed on home-based care and those with moderate to critical illness were admitted for care as per the WHO recommendations [14]. Mortality among hospitalized patients, especially patients who required management in the intensive care unit, remained relatively high globally. These patients often presented with complications such as respiratory failure, shock or multiorgan dysfunction, exacerbated by immune hyperactivation such as the cytokine storm [15]. To reduce mortality, especially among severe and critically ill patients, novel therapeutics such as tocilizumab were introduced. Studies indicated that tocilizumab use was associated with reduced mortality [16]. However, access to these novel treatments was limited in resource-constrained countries due to high demand and prohibitive costs [17].

Previous studies investigating the factors associated with poor outcomes among hospitalized COVID-19 patients have found that advanced age, male sex, and having comorbidities are significant predictors of mortality [18,19]. However, many of these studies in Africa analyzed data from the early phases of the pandemic. Recognizing the dynamic nature of COVID-19 and its varying impact over time, this study aims to identify and compare the nuances in predictors of mortality in each wave in Kenya.

## 2. Methods

### 2.1. Study Setting

We conducted a retrospective observation cohort study using medical records of laboratory-confirmed COVID-19 patients, hospitalized between 1 June 2020 and 30 August 2022 in Tigoni Level 4 Hospital which served as a dedicated COVID-19 treatment center for Kiambu County. Tigoni Hospital is situated within Kiambu County, Kenya, which is classified as peri urban. Kiambu County neighbors Nairobi County, which was the epicenter of the COVID-19 outbreak in Kenya. With a population of approximately 2.5 million people as per the 2019 national census, Kiambu County hosts significant commercial centers, contributing 5.7% to the national gross value add, ranking second to Nairobi County, and a per capita gross county product of approximately USD 2000 [20]. The study site, Tigoni Hospital, is classified as a Level 4 healthcare facility under the Kenya Essential Package for Health, boasts a bed capacity of 292 beds [21].

### 2.2. Study Participants

We included 1985 records of patients who tested positive for SARS-CoV-2 through either a reverse-transcription polymerase chain reaction or rapid antigen test and were diagnosed with moderate, severe, or critical COVID-19 symptoms. We excluded 57 records of patients who tested positive for SARS-CoV-2 but did not require admission to the hospital.

### 2.3. Ethical Approvals

The study received approval from the KNH-UoN Ethics and Review Committee under the approval number P287/04/2022. The approval date was 1 September 2022.

### 2.4. Data Collection

Demographic, clinical, laboratory, and outcome data were extracted from individual patient medical records. We employed a standardized data template on CommCare for data collection. Clinical data, including presenting symptoms, respiratory rate, temperature, and oxygen saturation (SaO2), were recorded at the time of initial admission and on subsequent follow-up days in hospital. The variables collected captured both static (non-time-varying) patient clinical profiles and dynamic (time-varying) factors throughout the admission period. The dynamic variables included the patient’s signs and symptoms, status of intensive care unit (ICU) admission, oxygen requirement, and changes in treatment modifications abstracted from the patient treatment records during the days in hospital. It is worth noting that due to limited laboratory and imaging capacity at the hospital, some radiological and laboratory data were frequently incomplete.

### 2.5. Outcomes of Interest and Statistical Analysis

The primary outcome of interest was in-hospital mortality. We conducted a descriptive analysis of patient characteristics based on their primary outcome status (alive or deceased). Numeric variables (age, average length of hospitalization, oxygen saturation) were treated as continuous variables if their density distribution plots indicated a normal distribution in either the natural or logarithmic scale. Otherwise, they were converted into categorical variables and presented as proportions. We analyzed the trends in admissions and mortality rates for each of the five distinct pandemic waves. The median length of hospital stay was calculated for both patients who were discharged and those who died during their hospital stay.

To investigate the effects of vaccination on patient outcomes, we created a subset of the data to represent the period when COVID-19 vaccines were made available in Kenya. This was estimated to be March 2021, when the first global rollout of COVAX vaccines was implemented in Africa [22]. We calculated and presented the overall survival probability using Kaplan–Meier survival curves, stratified by gender and ward admission (ICU or general ward). We further assessed the time-to-event analysis with death as the event of interest. The period was defined from the date of admission to the date of in-hospital death or discharge. We accounted for right-censored data, representing patients who were still hospitalized at the time of censoring, using Kaplan–Meier estimators.

### 2.6. Predictors of Mortality

To determine the patient characteristics associated with mortality over time and the effect size of each of the factors, we conducted our analysis in two steps. In the first step, we included static covariates; in the second step, we added dynamic covariates. Additionally, we examined how these factors differed across each of the five waves of the pandemic. The effect of selected covariates on the hazard of clinical outcomes was evaluated using univariable and multivariable Cox proportional hazard models. The proportional hazards assumptions were assessed using Schoenfeld residuals.

All statistical analyses were conducted using R version 4.3.2 with the Survival package version 3.5–7 [23,24]. The funders of this study had no involvement in the study design, data collection, analysis, interpretation, or reporting.

## 3. Results

### 3.1. Cohort Description

Between June 2020 and August 2022, Tigoni L4 Hospital admitted a total of 1985 patients who tested positive for SARS-CoV-2 virus. The diagnosis was based on either a reverse-transcription polymerase chain reaction or a rapid antigen test. All the patients had a comprehensive outcome available at the time of analysis.

Among the cohort, 557 individuals (28%) died during their hospitalization. The mean age of patients who died in hospital and those who were discharged was 66 and 54 years, respectively. The median length of hospital stay was 4 days (IQR: 1.0–8.0) for those who died and 9 days (IQR: 5.0–13.0) for patients discharged alive.

Among the cohort, the majority were male (54%); 1.3% were healthcare workers. Only 46 (2.8%) of all the patients had received at least one dose of the COVID-19 vaccine, with 998 (60%) remaining unvaccinated and 624 (37.4%) having an unknown vaccination status. Diabetes and cardiovascular diseases were the most prevalent comorbidities, accounting for 393 (36.9%) and 384 (36.1%) of the total comorbidities recorded, respectively. A total of 55 (2.8%) patients were admitted to the hospital’s ICU; 36 (65.5%) patients admitted to the ICU died. In addition, 1565 (79.1%) required oxygen at admission, with 1268 (73.8%) having low oxygen saturation below 90% (Table 1).

**Figure 1.**
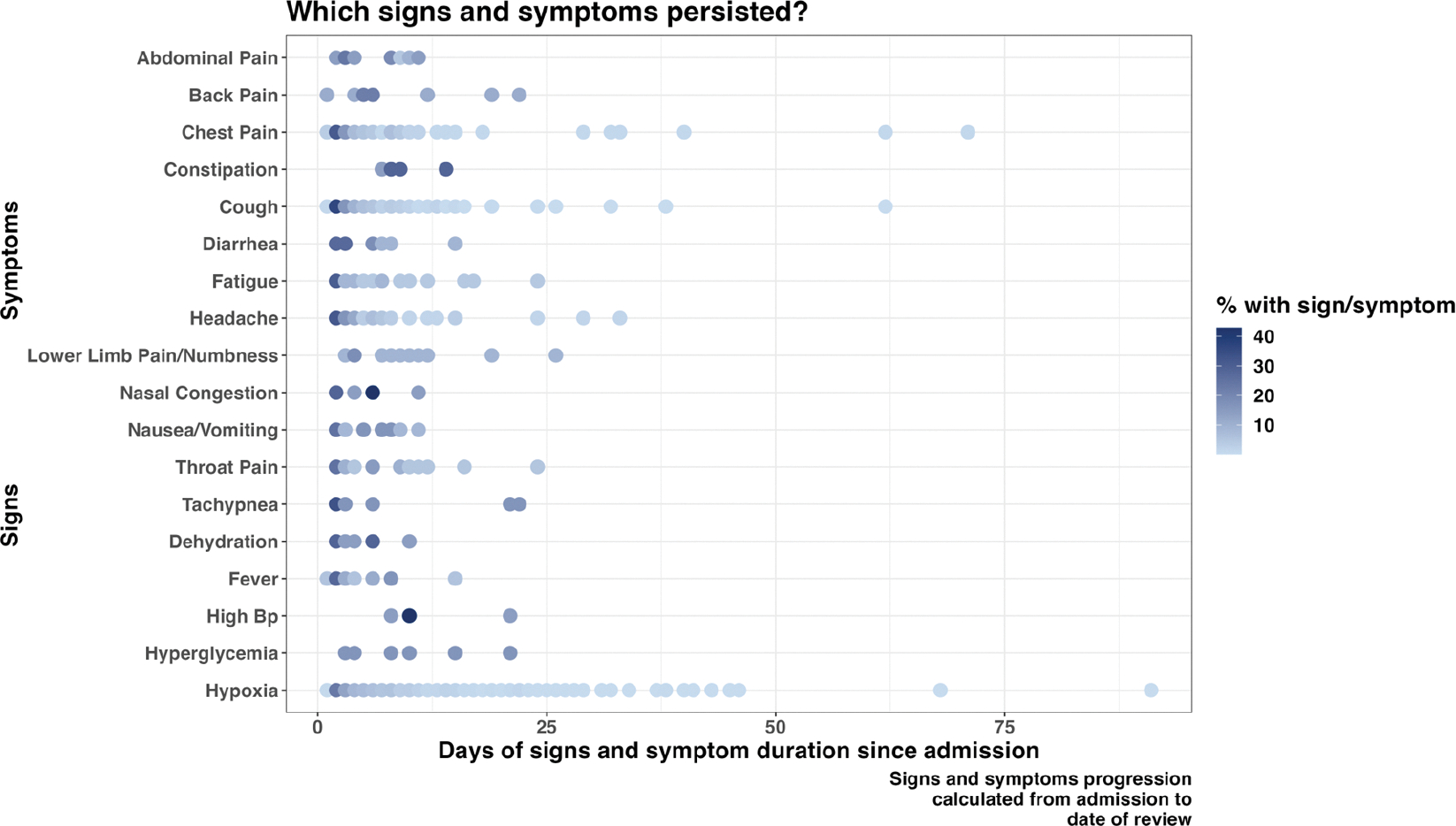
Progression of patients’ signs and symptoms from admission to the follow-up day(s).

**Figure 2.**
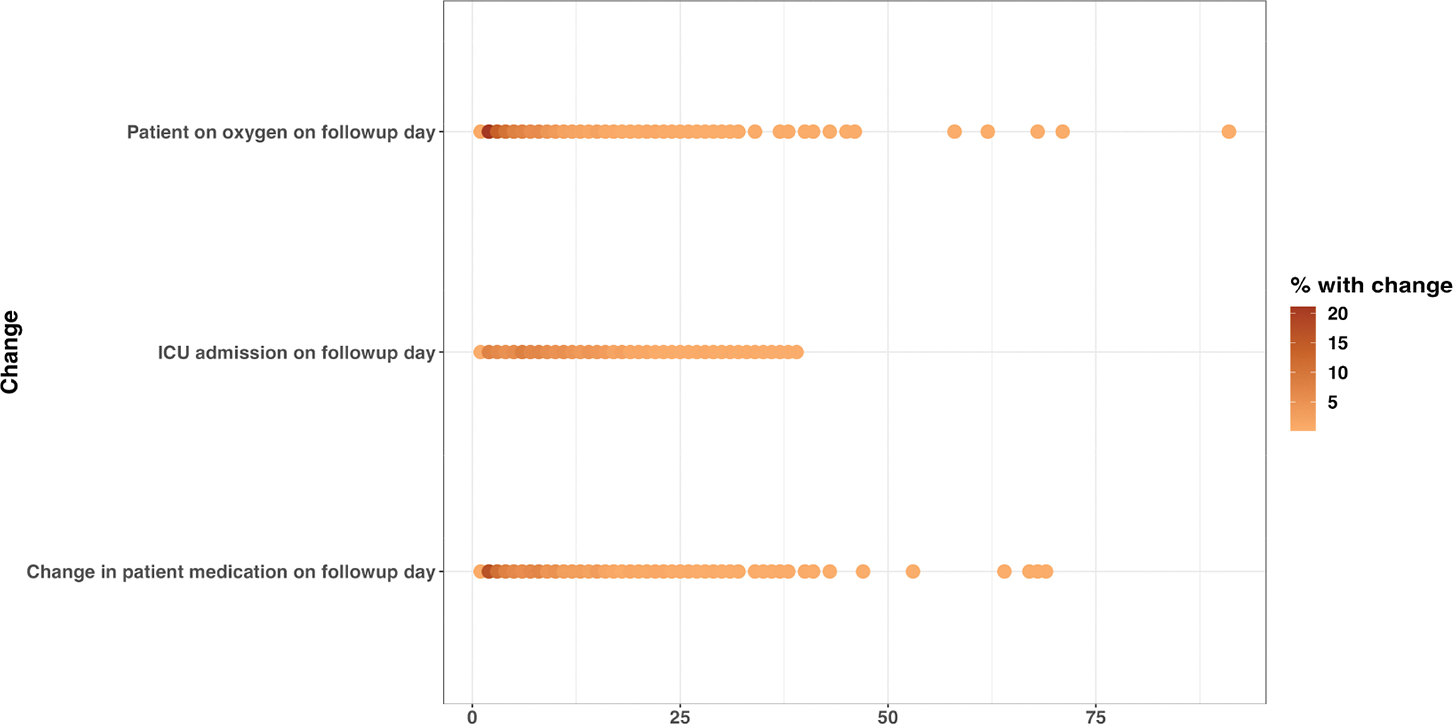
Change in patient clinical condition and management on follow-up day: oxygen requirement, ICU admission status, and medication change on follow-up date.

Cough and shortness of breath were the most predominant symptoms documented on admission, 1447 (28.8%) and 1380 (27.4%), respectively (Supplementary Table S1). Some signs and symptoms persisted in a majority of patients during the follow-up days, with hypoxia, chest pain, and cough being among the enduring manifestations. Furthermore, a proportion of patients still required adjustments in their treatment regimen more than two months after the first day of admission (Figures 1 and 2).

**Figure 3.**
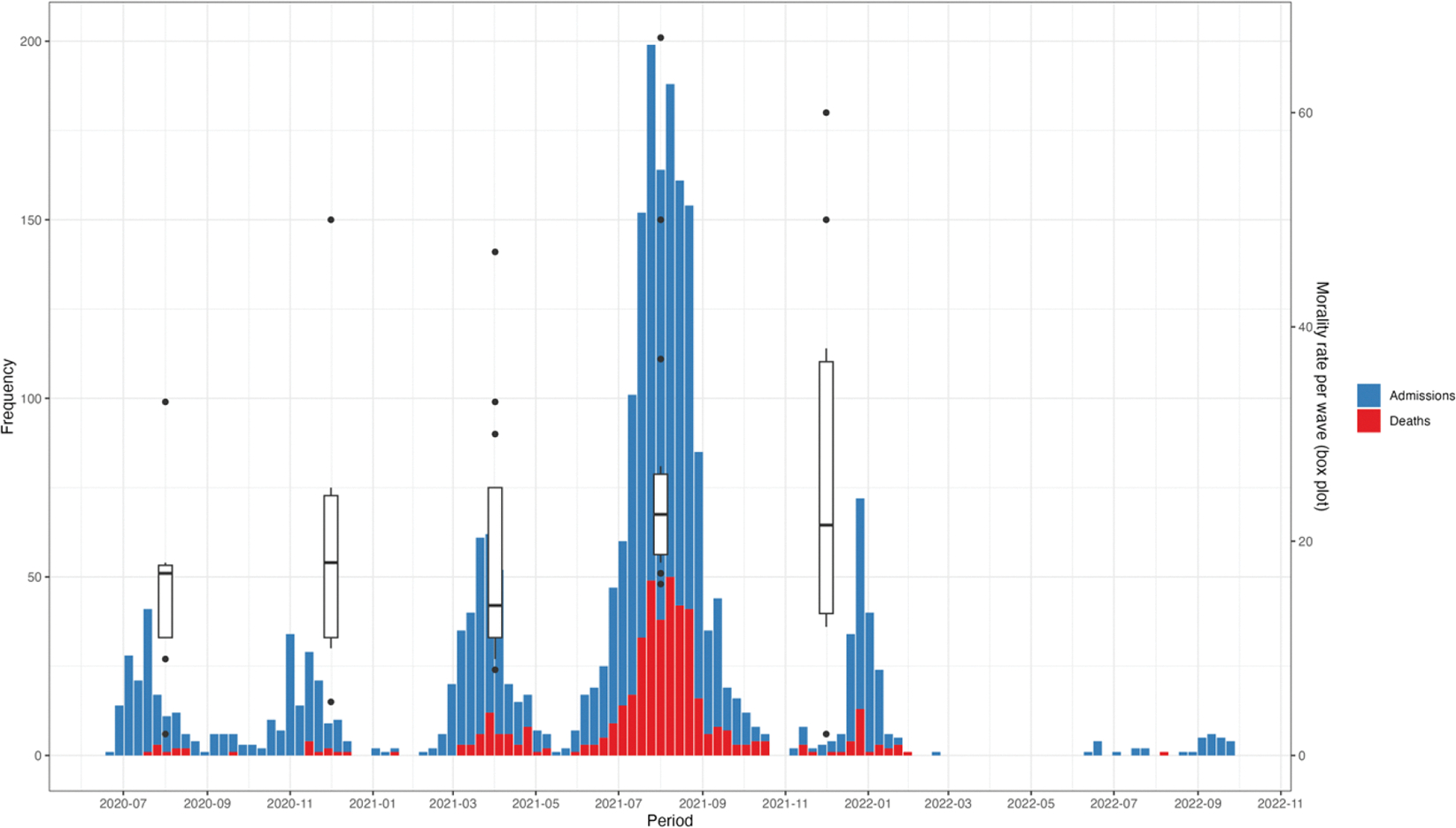
Trends in admissions, mortality, and mortality rates of hospitalized COVID-19 patients between July 2020 and November 2022. The x-axis represents the time period, with specific waves of the pandemic illustrated. The y1-axis (left) represents the number of admissions and deaths, while the y2-axis (right) represents the mortality rates per wave, illustrated by the box plots.

Peaks in hospitalization coincided with peaks in mortality (Figure 3). This pattern delineated across the five waves over the 26-month analysis period. Mortality rates also changed over time, following a similar trend to hospital admissions, with the highest (31%) and lowest (5%) mortality rates recorded during the fourth and first waves, respectively (Figure 3).

The overall one-year survival probability for the entire cohort was 43% (33%, 55%), with men having a lower survival probability compared to women, although the difference was not statistically significant (Supplementary Figure S1a). Furthermore, patients who were admitted to ICU had a lower survival probability compared to those who remained in the general ward (Supplementary Figure S1b).

### 3.2. Static Predictors of Mortality at Admission

Men were 1.38 times (CI: 1.13–1.7) more likely to decease during their hospital stay than women. Furthermore, patients admitted during the fourth and fifth waves were at a higher risk of death compared to those admitted during the first wave of the pandemic (HR: 3.13 [CI: 1.50–6.5] and 2.50 [1.11–5.60], respectively) (Supplementary Figure S2). The increased risk of death among men compared to women was more evident during the fourth wave and not significant in the other waves. Additionally, patients admitted with low oxygen saturation and either a positive or no history of chronic illness or pre-existing comorbidity were at a higher risk of death during the fourth wave compared to those admitted during the other four waves (Supplementary Figure S3).

### 3.3. Dynamic Predictors of Mortality During Follow-Up

**Figure 4.**
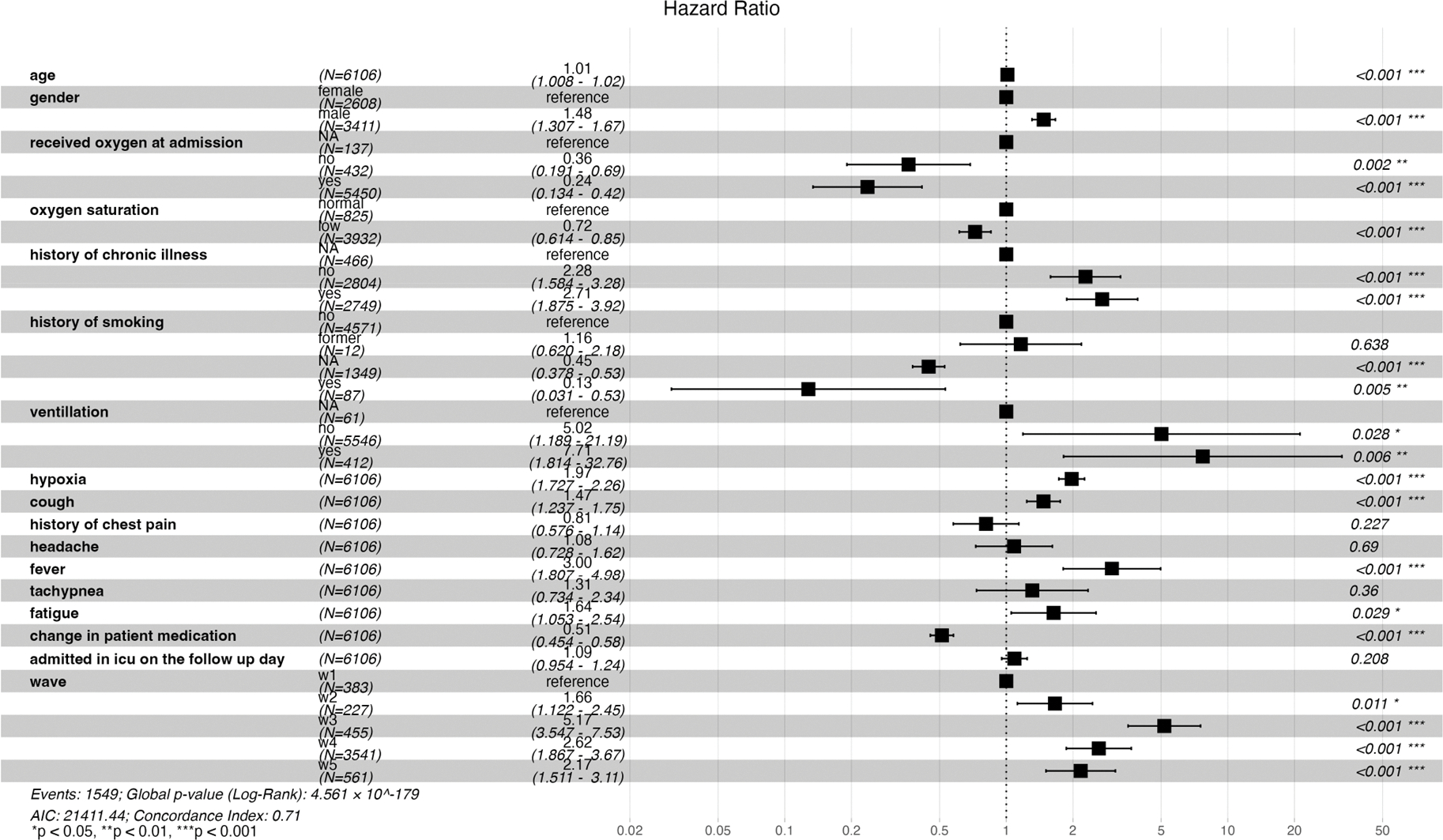
Forest plot illustrating both time- and non-time-varying predictors of mortality and the risk of mortality across the different waves.

An increase in age, male gender, no history, and a positive history of chronic illness were associated with statistically significant risk of mortality (HR: 1.01 [CI: 1.008–1.02], 1.48 [CI: 1.31–1.67], 2.28 [CI: 1.58–3.28], 2.71 [CI: 1.88–3.92], respectively). Patients who presented with persistent hypoxia, cough, fever, and fatigue had a significant increased risk of death (HR: 1.97 [CI: 1.73–2.26], 1.47 [CI: 1.24–1.75], 3.00 [CI: 1.81–4.98], 1.64 [CI: 1.05–2.54], respectively). Additionally, admission during waves two, three, four, and five was significantly associated with increased risk of mortality compared to wave one (HR: 1.66 [CI: 1.22–2.45], 5.17 [CI: 3.55–7.53], 2.62 [CI: 1.87–3.67], 2.17 [CI: 1.51–3.11], respectively). Patients who presented with low oxygen saturation at admission and received oxygen had a reduced risk in mortality (HR: 0.72 [0.61–0.85], 0.24 [0.13–0.42], respectively). A change in medication was associated with a 49% reduction in the risk of death (HR: 0.51 [CI: 0.45–0.58]) (Figure 4 below).

**Figure 5.**
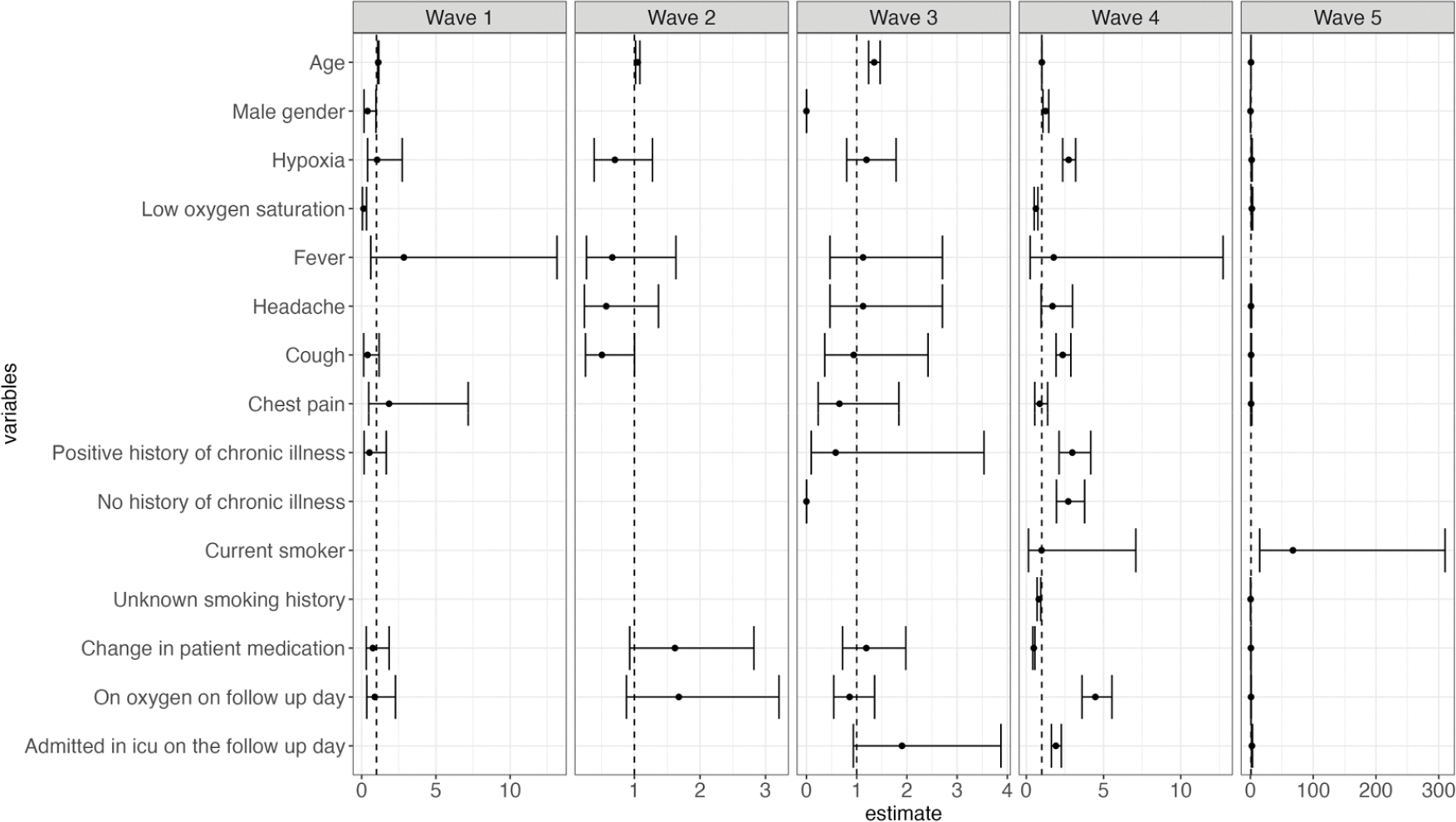
Forest plots illustrating both time- and non-time-varying predictors of mortality and the risk of mortality across each of the five different waves

During the third wave, older patients were at a higher risk of death, whereas male patients and those without a history of chronic illness had a reduced risk of mortality. Persistent hypoxia and cough, positive or no history of chronic illness, continuous use of oxygen, and ICU admission were associated with increased mortality during the fourth wave. Conversely, patients who presented with low oxygen saturation and whose treatment regimen was altered during their hospital stay had a reduced risk of death during wave four (Figure 5).

## 4. Discussion

Our comprehensive analysis of the five waves of the pandemic enabled us to delve into any subtle nuances of patient clinical profiles that could have contributed to an increased risk in mortality. The study reveals a crude in-hospital mortality rate of 28%, with the highest mortality observed during the fourth wave when the delta variant was predominant. The median length of hospitalization was shorter for patients who died in the hospital compared to those discharged alive. Men had a higher risk of death compared to women. Patients presenting with persistent hypoxia, cough, fever and fatigue faced a significantly higher risk of death. However, patients with low oxygen saturation at admission who received oxygen therapy showed a reduction in mortality risk. Furthermore, a change in the treatment regimen was also associated with a decreased mortality risk.

Reported hospital mortality rates vary by geographical region. Our study’s mortality rate was three times higher than that of a hospital study conducted in Spain [25]. Conversely, a UK study focusing on elderly patients reported a similar mortality rate of 27.2%, which was comparable to our findings given the mean age of patients in our study was 57.5 (SD: 18.3) years [26]. In contrast, a large hospital cohort study carried out in the USA reported a significantly lower mortality rate of 13.2% [27], while the mortality rates were comparable with other countries from the continent such as Tanzania, Ethiopia, and South Africa, which reported high mortality rates similar to our study (31.8%, 17.9%, 29.6%, respectively) [28–30]. In addition, we observed differences in mortality rates across the five waves, with a notable reduction in the risk of death between waves 3 and 5. The decrease in mortality in subsequent waves was also reported in an Italian study, which found a lower risk during the second wave compared to wave three [31]. The disparity in mortality between high- and low-income countries may be attributed to differences in healthcare infrastructure. Low- and middle-income countries often face challenges such as understaffing and poorly financed health systems, leading to worse survival outcomes [32]. In addition, access to novel therapeutics such as tocilizumab and early oxygenation strategies such as high flow nasal cannula and early prone positioning were more readily available in high-income countries [33]. Furthermore, the health workforce was significantly impacted over the course of the pandemic, with increased workload and staff shortages resulting from infections among healthcare workers and mounting exhaustion, which may have contributed to reduced quality of care.

A higher age was associated with increased mortality risk and men were 48% more likely to die compared to women. Several studies have reported similar increased risk of death among elderly patients, this has been associated with reduced ability to fight infections and a higher prevalence of comorbidities with advancing age [34–37]. The increased risk of death among men has been suggested to be due to higher prevalence of chronic conditions, smoking, and poorer immunological status in men compared to women [38]. Although our study lacked adequate data on smoking history, men are more frequent smokers than women and therefore more at risk of chronic respiratory conditions [39].

Persistent chest pain, cough, and hypoxia were common enduring symptoms, consistent with findings from other studies and associated with increased mortality risk [40,41]. Additionally, findings from other studies found approximately 31% to 61% of patients experienced multiple symptoms four weeks from their initial COVID-19 diagnosis [42]. These lingering symptoms also known as post-COVID-19 sequelae are related to the ability of SARS-CoV-2 to trigger an extensive inflammatory response [40]. A comprehensive study analyzing mortality predictors found that patients who presented with respiratory symptoms were 2.5 times more likely to die [43]. These findings underscore the critical need to monitor COVID-19 patients with prolonged respiratory signs and symptoms closely.

Supplemental oxygen was beneficial to hypoxic patients, although titration of oxygen was important to avoid overcorrection [44]. Patients who presented with low oxygen saturation had a 28% reduction in the risk of mortality, with a further reduction in risk by 76% upon receiving oxygen. Additionally, given the phenomenon of happy or silent hypoxia, described in patients with COVID-19, these findings underscore the need of consistent monitoring of oxygen saturation levels among COVID-19 patients [45]. While our study focused on hospitalized patients, these findings support those of a large South African study that found significantly lower morbidity rates in patients who monitored their oxygen saturation at home compared to those who did not [46].

Finally, advancements in medical care and treatment strategies throughout the pandemic led to improvements in patient outcomes. The use of new drugs such as corticosteroids, antivirals, and immunomodulatory drugs such as dexamethasone, remdesivir, and tocilizumab was shown to improve clinical outcomes [47–49]. Our study found a 49% reduction in mortality risk among patients whose treatment was modified during their hospital stay. The findings from our study could not explain the differences in mortality predictors by wave; therefore, there is a need for further research to gain better understanding of these variations.

## 5. Strengths

The study analyzed data from hospitalized patients across multiple waves of the COVID-19 pandemic, capturing how patient characteristics evolved with emerging variants and the predictors of mortality across the waves. Additionally, the inclusion of analysis of follow-up symptoms among these patients provides valuable insight into disease progression, key triggers of clinical deterioration, and indicators of post-COVID-19 sequelae underscoring the need for sustained post-discharge surveillance and long-term planning.

## 6. Limitations

Our study is limited by its reliance on data from a single healthcare facility, potentially limiting the generalizability of our findings. Furthermore, we could not determine the impact of COVID-19 vaccination on mortality due to incomplete vaccination data as well as variability in vaccine uptake in the population. Additionally, due to the manual recording of patient data, some of the patient information was often missing, especially laboratory test results.

## 7. Conclusions

In conclusion, our study underscores the dynamic nature of COVID-19 mortality risk and the need for continuous adaptation of clinical practices to mitigate these risks effectively. We found a significantly higher mortality rate compared to that reported in high-income countries, highlighting geographical disparities in healthcare resources. However, it is worth noting that the mortality risk decreased as the pandemic evolved, suggesting improvements in patient management over time. Crucially, the study highlights the benefits of routine oxygen saturation monitoring and appropriate oxygen supplementation in management of COVID-19 patients. These findings affirm that investments made in providing oxygen therapy were not only justified but essential, as they significantly improved patient outcomes and reduced mortality.

## Supplementary Material

Supplementary Table S1

**Supplementary Materials:** The following supporting information can be downloaded at: https://www.mdpi.com/article/10.3390/covid5060076/s1, Supplementary Table S1: Patients’ clinical symptoms by outcome; Supplementary Figure S1: Kaplan Meier plot on the survival probability by gender and ICU admission; Supplementary Figure S2: Forest plot illustrating the non-time varying predictors of mortality and the risk of mortality across the different waves; Supplementary Figure S3: Forest plots illustrating predictors of mortality at admission across each of the five waves.

## Figures and Tables

**Table 1.** Sociodemographic and clinical characteristics of hospitalized COVID-19 patients in Tigoni Level 4 Hospital, Kiambu County, Kenya.

Variable	Dead (N = 557)	Alive (N = 1428)	Total (N = 1985)

Age			
Mean (SD)	66.1 (17.0)	54.0 (17.8)	57.5 (18.3)
Range	0.0–109.0	0.000–106.0	0.0–109.0
Gender			
Female	235 (42.1%)	678 (47.5%)	913 (46.0%)
Male	322 (57.9%)	750 (52.5%)	1072 (54.0%)
Occupation	n = 556	n = 1420	n = 1976
Healthcare worker	1 (0.2%)	24 (1.7%)	25 (1.3%)
Non-healthcare worker	555 (99.8%)	1396 (98.3%)	1951 (98.7%)
Vaccinated	n = 534	n = 1134	n = 1668
No	280 (52.4%)	718 (63.3%)	998 (59.8%)
Yes	15 (2.8%)	31 (2.7%)	46 (2.8%)
Unknown	239 (44.8%)	385 (34.0%)	624 (37.4%)
ICU admission	n = 556	n = 1423	n = 1979
No	517 (93.0%)	1384 (97.3%)	1901 (96.0%)
Yes	36 (6.5%)	19 (1.3%)	55 (2.8%)
Unknown	3 (0.5%)	20 (1.4%)	23 (1.2%)
History of smoking	n = 556	n = 1423	n = 1979
Former	2 (0.4%)	1 (0.1%)	3 (0.2%)
No	464 (83.5%)	1071 (75.3%)	1535 (77.6%)
Yes	3 (0.5%)	9 (0.6%)	12 (0.6%)
Unknown	87 (15.6%)	342 (24.0%)	429 (21.6%)
Oxygen at admission	n = 556	n = 1423	n = 1979
No	22 (4.0%)	303 (21.3%)	325 (16.4%)
Yes	520 (93.5%)	1045 (73.4%)	1565 (79.1%)
Unknown	14 (2.5%)	75 (5.3%)	89 (4.5%)
Chronic conditions	n = 556	n = 1423	n = 1976
No	272 (48.9%)	728 (51.2%)	1000 (50.5%)
Yes	244 (43.9%)	541 (38.0%)	785 (39.7%)
Unknown	40 (7.2%)	154 (10.8%)	194 (9.8%)
Length of hospital stay			
Median (IQR)	4.0 (1.0–8.0)	9.0 (5.0–13.0)	9.652 (10.4)
Oxygen saturation	n = 463	n = 1256	n = 1719
Low (<90%)	416 (89.8%)	852 (67.8%)	1268 (73.8%)
Normal (>90%)	47 (10.2%)	404 (32.2%)	451 (26.2%)
Ventilatory support	n = 30	n = 15	n = 45
Invasive	1 (3.3%)	0 (0.0%)	1 (2.2%)
Non-invasive	29 (96.7%)	15 (100.0%)	44 (97.8%)

Abbreviations: SD: standard deviation; IQR: interquartile range.

## Data Availability

The data presented in this study are available on reasonable request from the corresponding author.
