# Supplementary material for: Mortality and Predictors of Mortality Among COVID-19 Patients in Kiambu County, Kenya
Source: COVID. Author manuscript; Available in PMC 2026 Feb 25. (PMC12931959; doi:10.3390/covid5060076)
Supplement: Supplementary Table S1 [file NIHMS2141849-supplement-Supplementary_Table_S1.pdf]

Supplementary Table S1: Patients' clinical symptoms by outcome.

Symptoms	Dead (N=1433)	Alive (N=3598)	Total (N=5031)
Chills	8 (0.6%)	17 (0.5%)	25 (0.5%)
Cough	413 (28.8%)	1034 (28.7%)	1447 (28.8%)
Diarrhea	17 (1.2%)	60 (1.7%)	77 (1.5%)
Dizziness	4 (0.3%)	18 (0.5%)	22 (0.4%)
Shortness of breath	464 (32.4%)	916 (25.5%)	1380 (27.4%)
Fatigue	79 (5.5%)	307 (8.5%)	386 (7.7%)
Fever	54 (3.8%)	199 (5.5%)	253 (5.0%)
Headache	68 (4.7%)	247 (6.9%)	315 (6.3%)
Loss of smell	4 (0.3%)	12 (0.3%)	16 (0.3%)
Loss of taste	2 (0.1%)	12 (0.3%)	14 (0.3%)
Muscle pains	79 (5.5%)	160 (4.4%)	239 (4.8%)
Nausea and vomiting	17 (1.2%)	26 (0.7%)	43 (0.9%)
Running nose	16 (1.1%)	68 (1.9%)	84 (1.7%)
Sore throat	30 (2.1%)	132 (3.7%)	162 (3.2%)
Weakness	56 (3.9%)	74 (2.1%)	130 (2.6%)
Other symptoms	122 (8.5%)	316 (8.8%)	438 (8.7%)

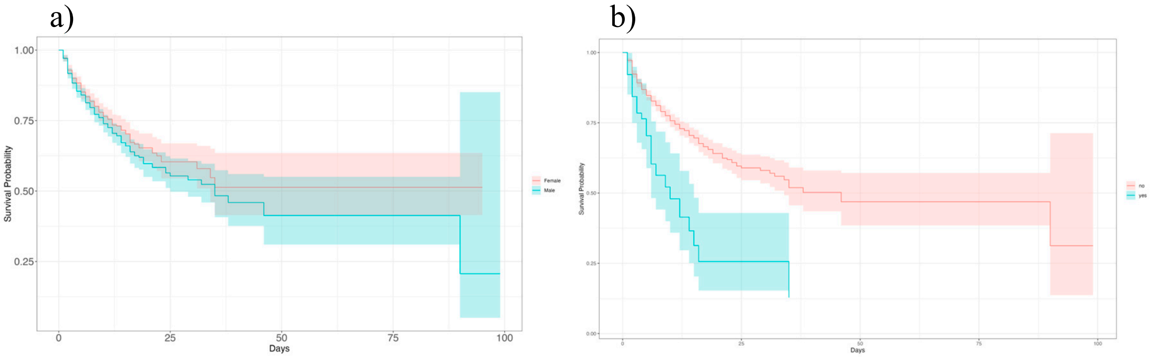

Supplementary Figure S1: Kaplan Meier plot on the survival probability of the study participants categorized by gender (a) and ICU admission (b).

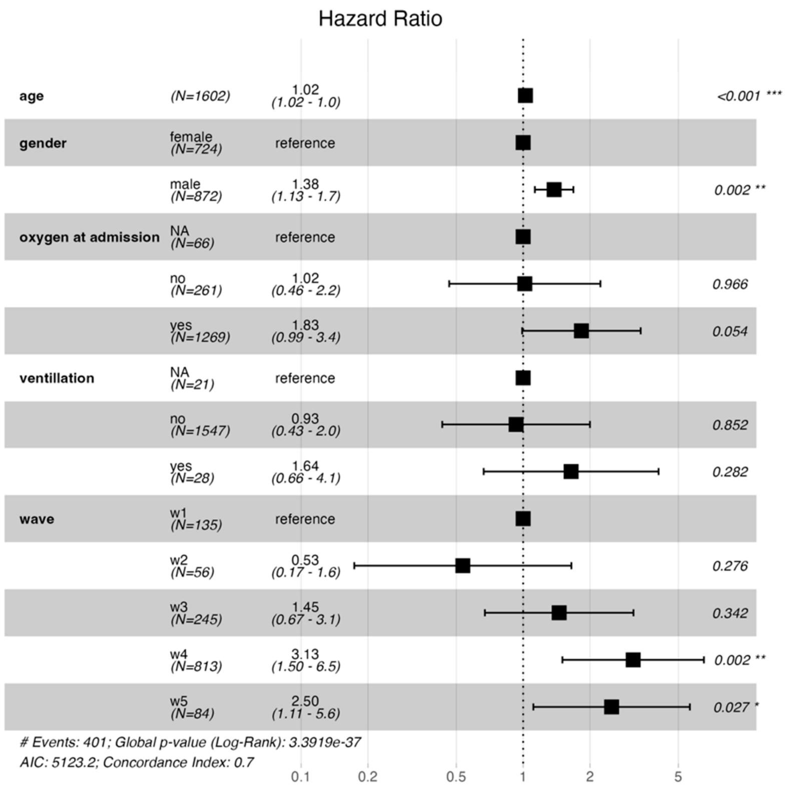

Supplementary Figure S2: Forest plot illustrating the non-time varying predictors of mortality and the risk of mortality across the different waves.

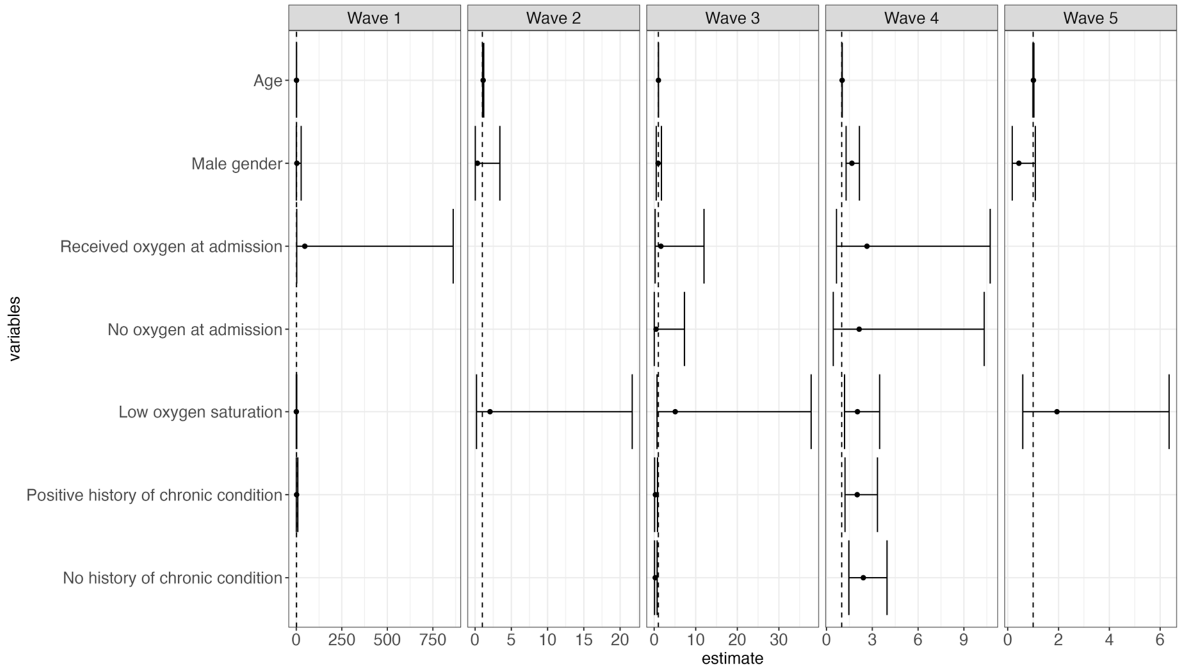

Supplementary Figure S3: Forest plots illustrating predictors of mortality at admission (non-time varying) across each of the five waves.
